# Osteonecrosis of the Jaw (ONJ) in Osteoporosis Patients: Report of Delayed Diagnosis of a Multisite Case and Commentary about Risks Coming from a Restricted ONJ Definition

**DOI:** 10.3390/dj5010013

**Published:** 2017-03-16

**Authors:** Mario Migliario, Giovanni Mergoni, Paolo Vescovi, Iolanda De Martino, Manuela Alessio, Luca Benzi, Filippo Renò, Vittorio Fusco

**Affiliations:** 1 Dental Clinic, Health Sciences Department, University of Eastern Piedmont “A. Avogadro”, 28100 Novara, Italy; mario.migliario@virgilio.it; 2 Oral Medicine and Laser Surgery Unit, University Center of Dentistry, Department of Medicine and Surgery, University of Parma, 43121 Parma, Italy; gmergon@gmail.com (G.M.); paolo.vescovi@unipr.it (P.V.); 3 Centro di Documentazione Osteonecrosi dei Mascellari, 15121 Alessandria, Italy; idemartino@ospedale.al.it (I.D.M.); vfusco@ospedale.al.it (V.F.); 4 Radiology Unit, Alessandria Hospital, 15121 Alessandria, Italy; lbenzi@ospedale.al.it; 5 Innovative Research Laboratory for Wound Healing, Health Sciences Department, University of Eastern Piedmont “A. Avogadro”, 28100 Novara, Italy; filippo.reno@med.uniupo.it; 6 Oncology Unit, Alessandria Hospital, 15121 Alessandria, Italy

**Keywords:** bisphosphonate, alendronate, osteoporosis, osteonecrosis of jaw, BRONJ, MRONJ, laser therapy

## Abstract

Osteonecrosis of the jaws (ONJ) in osteoporosis patients has been defined as rare, but the number of reported cases is increasing. We report a case of delayed ONJ diagnosis in a patient, who was being treated with alendronate, developing bone alterations both in maxilla and in mandible. Underestimation of ONJ incidence and missed or delayed ONJ diagnosis in osteoporosis patients might derive from lack of awareness of health providers as well as from an ONJ definition that is too restricted. The more recent definition of medication-related osteonecrosis of the jaws (MRONJ) released in 2014 by the American Association of Oral Maxillofacial Surgeons (AAOMS) accept fistula, besides bone exposure, as a major sign of disease, but it seems to be insufficient since it excludes all cases of ONJ disease without bone exposure. A new MRONJ definition is needed to avoid missing or delayed diagnosis.

## 1. Introduction

Osteonecrosis of the jaws (ONJ) has been reported in osteoporosis patients treated with oral bisphosphonates since 2004 [1], even as a minority in comparison with cases of cancer patients receiving intravenous bisphosphonates.

The first largely accepted definition of bisphosphonate-related osteonecrosis of the jaws (BRONJ) was released by the American Association of Oral Maxillofacial Surgeons (AAOMS) in 2007, and was defined as the presence of exposed necrotic bone in the maxillofacial region that has persisted for more than 8 weeks in patients with current or previous treatment with bisphosphonates, and no history of head and neck radiation to the jaws [2]. That definition was substantially acknowledged by a task force of American Society for Bone and Mineral Research (ASBMR) [3] and confirmed in 2009 by AAOMS [4].

In following years, occurrence of cases without bone exposure [5,6,7] questioned that definition [8,9,10], so that nowadays the ONJ definition is highly debated and controversial [11,12].

It is important to note that recently the BRONJ name has been converted by AAOMS to medication-related osteonecrosis of the jaw (MRONJ) to integrate the growing number of osteonecrosis cases involving the maxilla and mandible associated with another antiresorptive drug (denosumab) and targeted therapies [13]. In light of this latter position taken in the AAOMS paper, the disease definition was also slightly expanded to include cases with “*bone that can be probed through an intraoral or extraoral fistula in the maxillofacial region*” [13], previously classified as a prodromal “stage 0” status (out of the strict disease definition) in the 2009 classification [4]. Conversely, a panel of experts acknowledged by ASBMR [14] recently confirmed the 2007 definition without the AAOMS 2009 and 2014 amendments, enforcing the debate [11,12] about adjudication and time of diagnosis of ONJ cases without frank bone exposure, both among cancer patients and osteoporosis patients.

An unusual case of multisite ONJ without bone exposure in a patient who received alendronate therapy is here reported and discussed in light of possible delay of diagnosis due to a restricted ONJ definition.

## 2. Case Report

In February 2015, a 70-year-old female patient was referred by her medical practitioner to the Alessandria Hospital Osteonecrosis of the Jaws (ONJ) Multidisciplinary Team due to severe oral infections.

The woman reported to be affected by osteoporosis and had been receiving weekly treatment of 70 mg alendronate for 5 years. She denied any history of diabetes, severe cardiovascular disease, cancer, myeloma, head-neck radiotherapy, and facial trauma.

The patient complained of pain at the left maxilla and the right side of the mandible.

The recent dental history had started 15 months before, in November 2013, when the patient went to her dentist with the aim to restore the masticatory function compromised by a long period of neglect.

**Figure 1:**
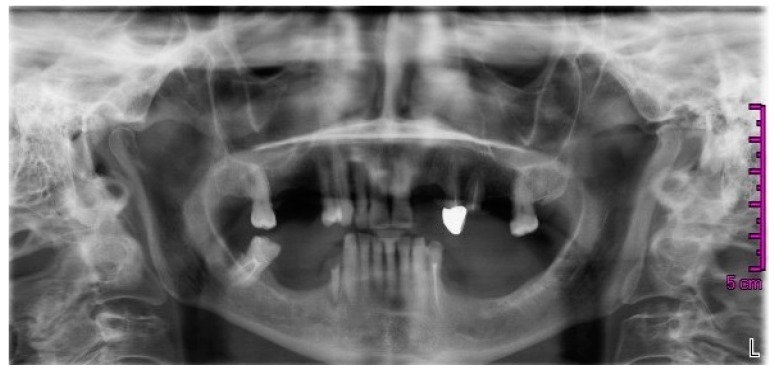
Orthopantomography dated November 2013.

The dentist, on the basis of the clinical and radiological evaluation (Figure 1), proposed the extraction of the roots of teeth 25 and 44 (ISO tooth-numbering system), root canal treatment of 27, the restoration with prosthetic crowns of the elements 27, 34, and 47, and the fabrication of a superior and an inferior partial removable prosthesis.

**Figure 2:**
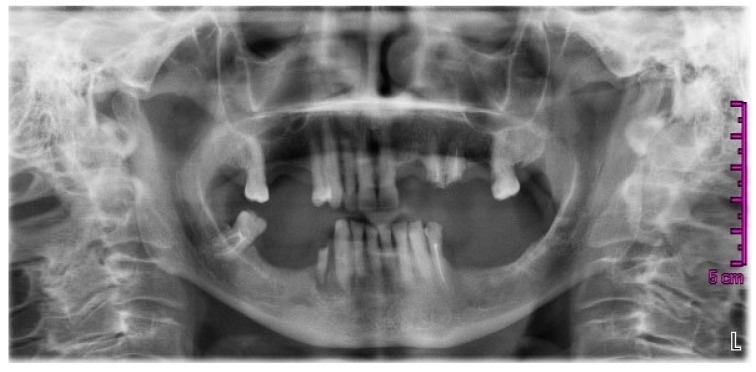
Orthopantomography dated March 2014.

In March 2014, the patient came back to the dentist practice with a new panoramic radiograph (Figure 2); this, according to the changed conditions (element 24 broken), varied the rehabilitation project, and the extraction of the roots of 24 and 25 were planned. An ill-defined area of radiolucency was present between the roots of 43 and 44 but no further investigations or considerations were made at that time.

The next month, the woman, with no prior antibiotic prophylaxis, underwent the extraction of the roots of 24 and 25 with local anesthesia; during the same appointment, she also underwent the extraction of the root 44, without local anesthesia because this root was extremely mobile; at the end of surgery, the dentist did neither sutured nor prescribed antibiotic therapy.

The following scheduled appointments were delayed due to the continuing pain and the lack of complete healing of the post-extractive sites.

**Figure 3:**
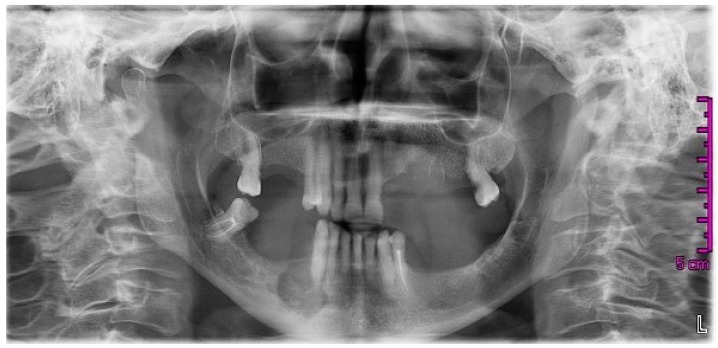
Orthopantomography dated October 2014, after extractions of teeth 24, 25, and 44. A wide radiolucent area involving the bone distal to 43 was present together with less-defined alterations in the upper left quadrant.

Six months later, in October 2014, the patient still presented with recurrence of abscesses on the second and fourth quadrant, a fistula in the region of 24/25, and third-degree mobility of the lower-right cuspid (43). The dentist requested a new radiograph (Figure 3) that showed a wide, circular radiolucent area involving the bone distal to 43 together with less-defined alterations in the upper left quadrant.

During the follow-up in December 2014, on the basis of what had been observed in the oral cavity and on the panoramic radiograph, the practice dentist hypothesized the presence of bone sequestrum in the upper-left bicuspid region and of a cystic lesion englobing the root of 43. He prescribed amoxicillin (1 g, 1 tablet twice a day for 6 days) and chlorhexidine (0.20%) oral rinses. At the next check-up, objective and subjective symptoms were still present and the dentist proposed a surgical revision of post-extraction sites.

**Figure 4:**
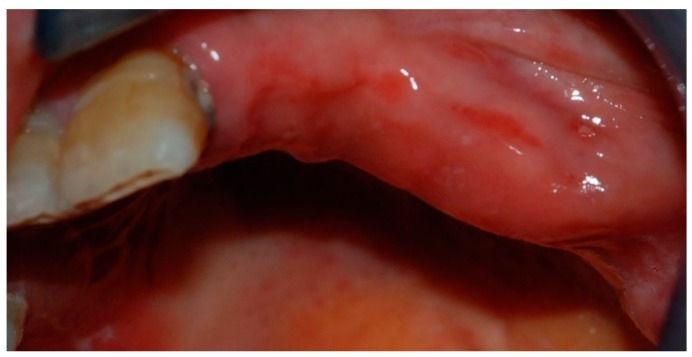
Inflamed gingival tissue at the upper-left maxillary region.

In February 2015, the patient decided to have a second opinion, and her medical practitioner asked for evaluation at the Osteonecrosis of Jaws (ONJ) Multidisciplinary Group at Alessandria Hospital. At clinical inspection, the upper-left premolar area showed granulating tissue with underlying apparent necrotic bone upon probing (Figure 4). 

**Figure 5:**
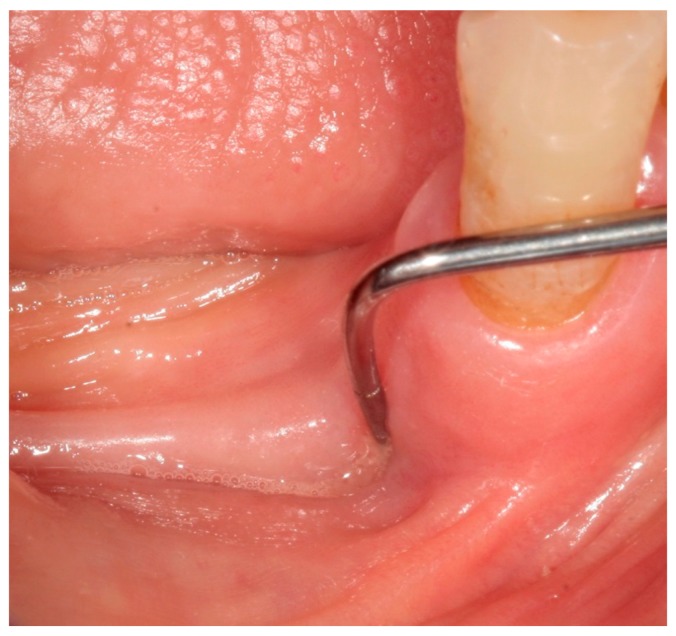
Fistula that probes to bone distal to 43.

In the right mandible, a hard-elastic painful swelling and a fistula distal to 43 were observed (Figure 5).

**Figure 6:**
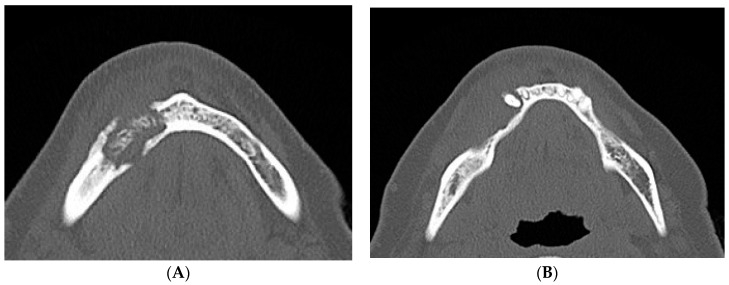
(**A**,**B**) Computed tomography (CT) scan dated February 2015, which showed a large sequestrum in right mandible along with erosion of the buccal and lingual cortical plates.

Hence, on the basis of the recent dental history, a diagnosis of multifocal non-exposed MRONJ was suspected and alendronate intake was suspended. After the first visit, a computed tomography (CT) scan of the jaws, without iodine contrast, was performed and it showed that on the right side of the mandible was an osteolytic area 20 mm in diameter, extended in the region of the premolars, englobing the root of the lower-right cuspid, and containing bone fragments. This lesion determined swelling of the buccal mandibular cortical, which appeared also eroded both on the lingual and buccal side; the adjacent spongious bone and the periosteum on the buccal side appeared thickened (Figure 6).

**Figure 7:**
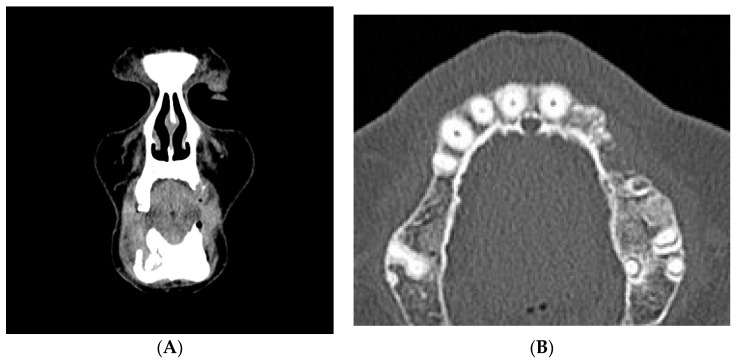
(**A**,**B**) CT scan dated February 2015. Coronal and axial cuts showing erosion of the left maxillary alveolar bone.

In the left maxillary, a large area of bone rarefaction and erosion extending from the area of cuspid to second premolar was observed (Figure 7).

After the CT scan, the patient was referred to the University Oral Care Center in Novara, which was nearer to her hometown. Here the clinicians, confirming the diagnosis of MRONJ, prescribed amoxicillin + clavulanic acid (1 g, 1 tablet every 12 h), metronidazole (250 mg, 2 tablets every 12 h), and a 0.20% chlorhexidine oral rinse every 12 h.

The patient was proposed to undergo surgical intervention in order to remove the necrotic bone which was causing the recurrent abscesses. She was referred to a third-level center for ONJ, the Unit of Oral Pathology and Laser-assisted Oral Surgery of the University of Parma, which specialized in laser-assisted treatments of MRONJ. Here the patient accepted to undergo surgical operation with local anesthesia. The surgery consisted of the osteotomy of the right alveolar ridge of mandible and vaporization of osteonecrotic area with Er:YAG laser; extraction of 42, 43, and 34; and surgical revision of the upper-left premolar region.

Before surgery, the patient received antibiotic therapy (the previous antibiotic regimen was substituted with ciprofloxacin (500 mg, 1 tablet every 12 h) because a bacterial strain of *Serratia marcescens* resistant to amoxicillin plus clavulanate and metronidazole was isolated by means of cultural exam) and sessions of low-level laser therapy (LLLT) with a diode device (Giotto^®^, Dental Medical Technologies SRL, Lissone, Italy; parameters: 980 nm, optical fiber of 600 microns in diameter, 1 W continuous wave, 120 s, 3 times a week for 4 weeks).

In June 2015, the planned surgical intervention was performed without any intraoperative complications.

She was discharged with the indication to continue antibiotics and LLLT for 21 days until the removal of sutures and to apply chlorhexidine gel 1% on surgical wounds twice daily.

Four bone specimens collected from the mandible during surgery were subjected to histopathological examination: the one including dental elements 43 and 42 appeared macroscopically grey, of impaired consistency, and irregular surface with histopathological signs of osteonecrosis. The two bone chips collected mesially and distally near the osteolytic lesion and the one taken nearby the extractive socket of 34 consisted of normal bone.

Seven days after surgery, the patient had a perimandibular hematoma but the postsurgical wound was fully closed. Sutures were removed after 3 weeks. At this time, the patient ended the antibiotic therapy but continued the application of local antiseptics. 

**Figure 8:**
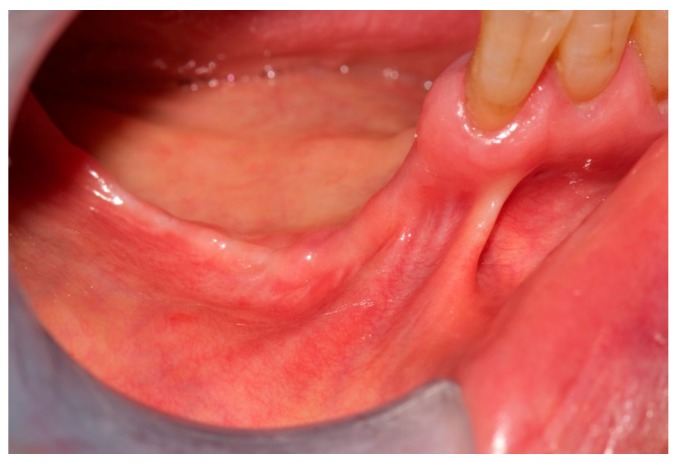
Three-month follow-up after surgical treatment for medication-related osteonecrosis of the jaws (MRONJ): complete clinical healing was achieved both in mandible and maxilla.

**Figure 9:**
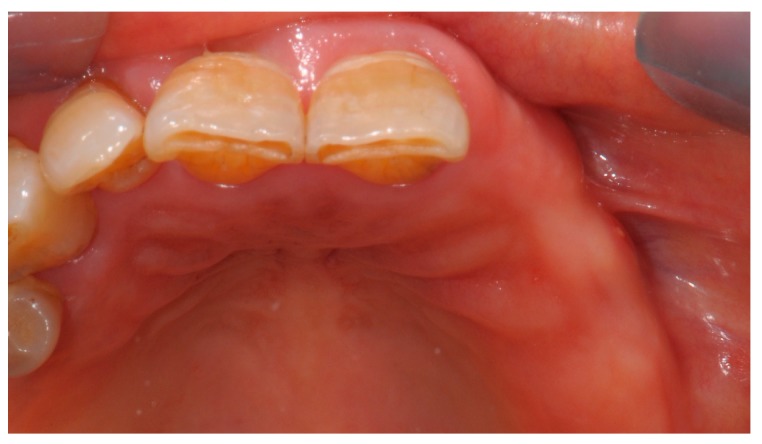
Three-month follow-up after surgical treatment for MRONJ: complete clinical healing was achieved both in mandible and maxilla.

A 3-month follow-up consisting of oral inspection and professional dental prophylaxis was scheduled. The woman neither present exposed necrotic bone in the oral cavity nor complained of any symptoms (Figure 8 and Figure 9).

During the follow-up period, it was suggested to the patient that teeth 27 and 48 be extracted. The elements were non-restorable and carried the potential risk of causing infection and triggering new foci of bone osteonecrosis.

Tooth extractions were performed in February 2016 at Parma University. Antibiotic therapy (amoxicillin + clavulanic acid, 1 g tablets, 1 tablet every 12 h; and metronidazole, 250 mg tablets, 2 tablets every 12 h) was started 3 days before the intervention and continued for 2 weeks after it. Twelve sessions of LLLT were performed before and after the intervention.

After 7 days, the patient reported mild bleeding from the mandibular socket; after 10 days, at the same site, a small mucosal dehiscence appeared. After 18 days, the dehiscence resolved with local applications of 1% chlorhexidine gel and a complete reepithelization of the extractive sockets was achieved 4 weeks after tooth extractions.

**Figure 10:**
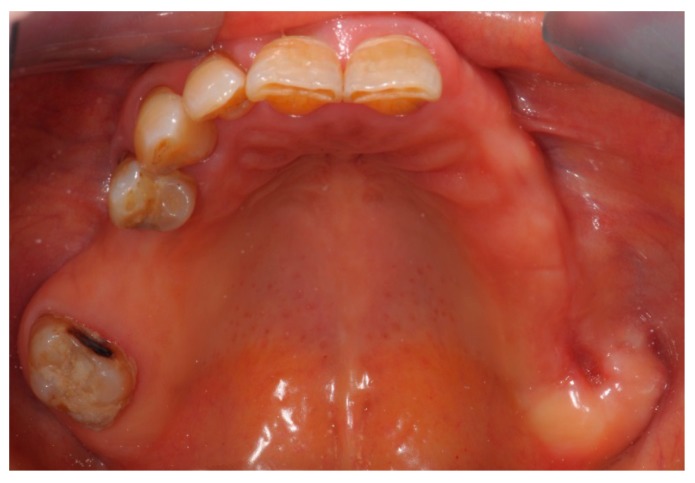
October 2016 follow-up visit: the oral mucosa showed no dehiscence in the areas of extractions and no other symptoms occurred.

**Figure 11:**
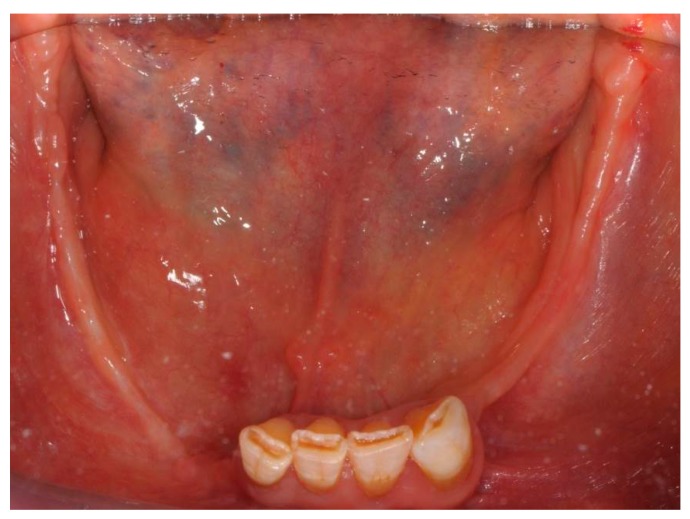
October 2016 follow-up visit: the oral mucosa showed no dehiscence in the areas of extractions and no other symptoms occurred.

**Figure 12:**
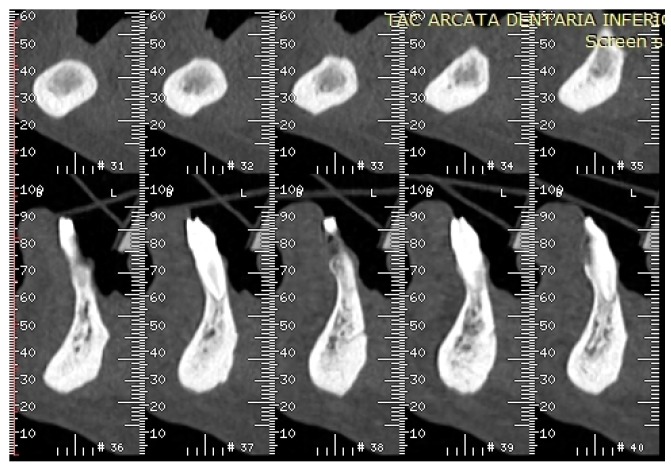
CT scan dated October 2016 showed no erosion of the mandibular buccal and lingual cortical bone.

**Figure 13:**
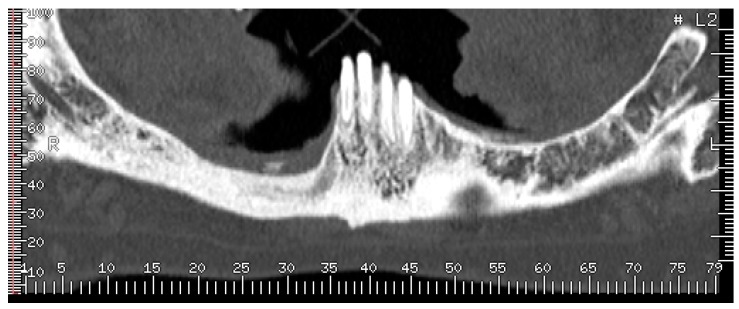
CT scan dated October 2016 showed no erosion of the mandibular buccal and lingual cortical bone.

At the oral inspection carried out in October 2016, the oral mucosa showed no dehiscence (Figure 10 and Figure 11) in the areas of extractions, and mandible CT scans without iodine contrast revealed no erosion of the mandibular cortical bone (Figure 12 and Figure 13). The upper arch of the patient was rehabilitated with a removable partial denture with cast clasps on elements 17, 14, and 21, and the lower arch with a removable partial denture with cast claps on elements 33 and 41. 

## 3. Discussion

### 3.1. Origin of ONJ

Development of drug-related ONJ has been reported to occur spontaneously or after tooth extraction, dental procedures, and infections, especially in subjects with poor oral hygiene [1]. Pathogenesis of bisphosphonate-related ONJ is believed to be multifactorial, involving altered bone turnover, infection, and altered immune system, but also inhibition of angiogenesis by bisphosphonates and other mechanisms [15,16,17,18].

### 3.2. ONJ Related to Alendronate and Other Drugs Administered for Osteoporosis

In the literature reports, ONJ in osteoporosis patients has been defined as rare on the basis of some trials and reviews [3,4,14], but the number of reported cases is increasing. ONJ case reports and case series in osteoporosis patients have been published worldwide after oral bisphosphonates (alendronate, risedronate, ibandronate), intravenous bisphosphonates (i.e., yearly 5 mg intravenous zoledronic acid, or intravenous ibandronate at 3 mg dose every 3 months), as well as subcutaneous denosumab (at 60 mg dose every 6 months). Even if these are probably a minority of cases actually observed in real life, hundreds of patients were included in large drug surveillance reports, in spite of a generalized poor attitude of physicians and dentists with regard to registering drug side effects [19,20,21,22,23,24].

### 3.3. Learning from the Case

The case herein described clearly questions the classical bisphosphonates-related ONJ definition diffused by AAOMS [2,4] and ASBMR [3,14] taskforces, based only on clinical observation of a long-duration bone exposure, and, above all, it questions the possibility of an early diagnosis on the basis of such a restricted definition.

At October 2014, objective status, 6 months after left maxilla and right mandible region extractions (probably due to already-underlying ONJ disease) revealed only a fistula. According to the classical BRONJ definition, the patient should have been only followed until appearance of a frank exposed-bone area and then furthermore observed for 8 weeks to reach a definitive ONJ diagnosis [2,3,4]. Furthermore, according to the same AAOMS staging system [4], in spite of recurrent abscesses and bone involving disease at the Rx exam, only an alerted observer might have classified the case into “stage 0” [4], a limbus state of “suspected” or “prodromal” disease. At that same time, the case could have been considered as a case of mono-site MRONJ according to the slightly expanded 2014 AAOMS definition [13], due to presence of a sequestrum in the left maxilla (but not yet in the right side of the mandible), and only after a further 2 months of observation. Consequently, even with this more recent definition, this kind of diagnosis was clearly late and limited to one site.

We do not agree with this “wait and see” attitude in the presence of a clinical and imaging suspect BRONJ diagnosis. Consequently, after the first observation by a multidisciplinary ONJ team, the patient underwent a CT scan and then appropriate medical and surgical therapy, including laser therapy.

In our opinion, the correct diagnosis might have been made much earlier and more appropriately (i.e., multisite instead of mono-site), if appropriate imaging (CT scan) had been performed months before, when signs and symptoms (recurring abscesses on the second and fourth quadrant; a fistula in the left maxilla region; high degree mobility of the lower-right canine) and the alendronate treatment history could well pose the suspect of a multisite “non-exposed” ONJ.

On the other hand, this case reinforces the importance of a lack of full ONJ awareness amongst health care professionals, reported in several papers [25,26,27,28,29], as a main determinant reason for possible diagnosis delay. A careful drug history of the patient and the suspect of ONJ in cases of unspecific oral disease signs and symptoms are cornerstones to reach an earlier diagnosis. However, imaging tools, particularly CT scan, are fundamental, in our opinion, to reach this goal, as shown by the multicenter MISSION study [30,31]. Furthermore, the cone beam computed tomography (CBCT) has been recently introduced with an advantage of lower X-ray dosage [32].

After intensive medical and surgical care, we observed resolution of mucosal break and cessation of repeated infections both in maxilla and mandible sites. The delay of diagnosis and treatment fortunately had no effect on the prognosis of this case, although with evident worsening of quality of life during the time between symptom onset (spring 2014) and treatment (March 2015).

However, we know that life-threatening or lethal drug-induced ONJ cases have been observed not only in cancer and myeloma [33,34,35,36] but also in osteoporotic patients [37,38,39,40] due to infectious complications; consequently, a diagnosis delay has to be avoided as much as possible.

### 3.4. Frequency of ONJ in Osteoporosis Patients after Bisphosphonates or Denosumab: Possible Underestimation and Late Diagnosis

In 2004–2005, after first literature papers and congress reports, national drug regulatory agencies launched alerts about ONJ risk, inducing higher awareness about the disease. The number of ONJ cases in noncancer patients was initially a minority of published cases, and the ONJ risk in osteoporosis patients (prevalently treated with oral bisphosphonates) appeared immediately lower than in cancer and myeloma patients receiving large amounts of high-dose intravenous bisphosphonates [1]. However, an increasing number of ONJ cases in osteoporosis and rheumatoid arthritis patients were observed in later years after receiving bisphosphonates therapy [31,41,42,43,44]. The same observation was more recently reported for denosumab: few ONJ cases were reported in trials, but an increasing number of cases are published on osteoporosis patients after more or less prolonged treatment with denosumab alone or bisphosphonates switched to denosumab [40,45,46,47,48].

In real life, ONJ cases after osteoporosis treatment do not seem as rare as one could expect from the results of few published trials. A considerable amount of ONJ cases in patients without cancer or myeloma history are commonly observed in most large oral care and maxillofacial surgery centers. Some multicenter surveys among dental care units and maxillofacial surgery departments that collected dozens of ONJ cases in noncancer patients have been reported worldwide, sometimes outnumbering ONJ cases in cancer patients [20]. Whether the number of cases is underestimated by a too-restricted ONJ definition, including those cases observed in cancer [12], is still to be fully explored.

## 4. Conclusive Remarks

Clinicians involved in the care of osteoporosis patients receiving antiresorptive agents (i.e., bisphosphonates and/or denosumab) should be aware that ONJ is uncommon but not rare. In the presence of suspected signs and symptoms (such as abscesses, fistulae, unexplained or sudden tooth mobility, perimplantitis, etc.) together with an antiresorptive drug history, further bone-imaging exams (such as CT scan or cone beam CT scan) should be considered to ascertain early signs of an osteonecrotic process and to avoid a delayed ONJ diagnosis.

## References

[1] Ruggiero S.L. (2009). Bisphosphonate-related osteonecrosis of the jaw (BRONJ): Initial discovery and subsequent development. J. Oral Maxillofac. Surg..

[2] Advisory Task Force on Bisphosphonate-Related Ostenonecrosis of the Jaws, American Association of Oral and Maxillofacial Surgeons (2007). American Association of Oral and Maxillofacial Surgeons Position Paper on Bisphosphonate-Related Osteonecrosis of the Jaws. J. Oral Maxillofac. Surg..

[3] Khosla S., Burr D., Cauley J., Dempster D.W., Ebeling P.R., Felsenberg D., Gagel R.F., Gilsanz V., Guise T., Koka S. (2007). Bisphosphonate-associated osteonecrosis of the jaw: Report of a task force of the American Society for Bone and Mineral Research. J. Bone Miner. Res..

[4] Ruggiero S.L., Dodson T.B., Assael L.A., Landesberg R., Marx R.E., Mehrotra B., American Association of Oral and Maxillofacial Surgeons (2009). American Association of Oral and Maxillofacial Surgeons Position Paper on Bisphosphonate-Related Osteonecrosis of the Jaw—2009 Update. J. Oral Maxillofac. Surg..

[5] Junquera L., Gallego L. (2008). Non exposed bisphosphonate-related osteonecrosis of the jaws: Another clinical variant?. J. Oral Maxillofac. Surg..

[6] Mawardi H., Treister N., Richardson P. (2009). Sinus tracts—An early sign of bisphosphonate-associated osteonecrosis of the jaws?. J. Oral Maxillofac. Surg..

[7] Fedele S., Porter S.R., D’Aiuto F., Aljohani S., Vescovi P., Manfredi M., Arduino P.G., Broccoletti R., Musciotto A., Di Fede O. (2010). Nonexposed variant of bisphosphonate-associated osteonecrosis of the jaw: A case series. Am. J. Med..

[8] Colella G., Campisi G., Fusco V. (2009). American Association of Oral and Maxillofacial Surgeons position paper: Bisphosphonate-Related Osteonecrosis of the Jaws—2009 update: The need to refine the BRONJ definition. J. Oral Maxillofac. Surg..

[9] Bedogni A., Fusco V., Agrillo A., Campisi G. (2012). Learning from experience. Proposal of a refined definition and staging system for bisphosphonate-related osteonecrosis of the jaw (BRONJ). Oral Dis..

[10] Schiodt M., Reibel J., Oturai P., Kofod T. (2014). Comparison of non-exposed and exposed bisphosphonate-induced osteonecrosis of the jaws: A retrospective analysis from the Copenhagen cohort and a proposal for an updated classification system. Oral Surg. Oral Med. Oral Pathol. Oral Radiol..

[11] Otto S., Marx R.E., Tröltzsch M., Ristow O., Ziebart T., Al-Nawas B., Groetz K.A., Ehrenfeld M., Mercadante V., Porter S. (2015). Comments on “diagnosis and management of osteonecrosis of the jaw: A systematic review and international consensus. J. Bone Miner.Res..

[12] Fusco V., Bedogni A., Addeo A., Campisi G. (2016). Definition and estimation of osteonecrosis of jaw (ONJ), and optimal duration of antiresorptive treatment in bone metastatic cancer patients: supplementary data from the denosumab extension study?. Support. Care Cancer.

[13] Ruggiero S.L., Dodson T.B., Fantasia J., Goodday R., Aghaloo T., Mehrotra B., O’Ryan F. (2014). American Association of Oral and Maxillofacial Surgeons, American Association of Oral and Maxillofacial Surgeons position paper on medication-related osteonecrosis of the jaw—2014 update. J. Oral Maxillofac. Surg..

[14] Khan A.A., Morrison A., Hanley D.A., Felsenberg D., McCauley L.K., O’Ryan F., Reid I.R., Ruggiero S.L., Taguchi A., Tetradis S. (2015). International Task Force on Osteonecrosis of the Jaw, Diagnosis and management of osteonecrosis of the jaw: A systematic review and international consensus. J. Bone Miner. Res..

[15] Allen M.R., Burr D.B. (2009). The pathogenesis of bisphosphonate-related osteonecrosis of the jaw: So many hypotheses, so few data. J. Oral Maxillofac. Surg..

[16] Kühl S., Walter C., Acham S., Pfeffer R., Lambrecht J.T. (2012). Bisphosphonate-related osteonecrosis of the jaws—A review. Oral Oncol..

[17] Campisi G., Fedele S., Fusco V., Pizzo G., Di Fede O., Bedogni A. (2014). Epidemiology, clinical manifestations, risk reduction and treatment strategies of jaw osteonecrosis in cancer patients exposed to antiresorptive agents. Future Oncol..

[18] Vermeer J.A., Renders G.A., Everts V. (2016). Osteonecrosis of the Jaw—A Bone Site-Specific Effect of Bisphosphonates. Curr. Osteoporos. Rep..

[19] Edwards B.J., Gounder M., McKoy J.M., Boyd I., Farrugia M., Migliorati C., Marx R., Ruggiero S., Dimopoulos M., Raisch D.W. (2009). Pharmacovigilance and reporting oversight in US FDA fast-track process: bisphosphonates and osteonecrosis of the jaw. Lancet Oncol..

[20] Lee J.K., Kim K.W., Choi J.Y., Moon S.Y., Kim S.G., Kim C.H., Kim H.M., Kwon Y.D., Kim Y.D., Lee D.K. (2013). Bisphosphonates-related osteonecrosis of the jaw in Korea: A preliminary report. J. Korean Assoc. Oral Maxillofac. Surg..

[21] De Boissieu P., Kanagaratnam L., AbouTaam M., Roux M.P., Dramé M., Trenque T. (2014). Notoriety bias in a database of spontaneous reports: The example of osteonecrosis of the jaw under bisphosphonate therapy in the French national pharmacovigilance database. Pharmacoepidemiol. Drug. Saf..

[22] Parretta E., Sottosanti L., Sportiello L., Rafaniello C., Potenza S., D’Amato S., González-González R., Rossi F., Colella G., Capuano A. (2014). Bisphosphonate-related osteonecrosis of the jaw: An Italian post-marketing surveillance analysis. Expert Opin. Drug Saf..

[23] Schiodt M., Larsson Wexell C., Herlofson B.B., Giltvedt K.M., Norholt S.E., Ehrenstein V. (2015). Existing data sources for clinical epidemiology: Scandinavian Cohort for osteonecrosis of the jaw—Work in progress and challenges. Clin. Epidemiol..

[24] De Boissieu P., Gaboriau L., Morel A., Trenque T. (2016). Bisphosphonate-related osteonecrosis of the jaw: Data from the French national pharmacovigilance database. Fundam. Clin. Pharmacol..

[25] López-Jornet P., Camacho-Alonso F., Molina-Miñano F., Gomez-Garcia F. (2010). Bisphosphonate-associated osteonecrosis of the jaw. Knowledge and attitudes of dentists and dental students: A preliminary study. J. Eval. Clin. Pract..

[26] Yoo J.Y., Park Y.D., Kwon Y.D., Kim D.Y., Ohe J.Y. (2010). Survey of Korean dentists on the awareness on bisphosphonate-related osteonecrosis of the jaws. J. Investig. Clin. Dent..

[27] Osta L.E., Osta B.E., Lakiss S., Hennequin M., Osta N.E. (2015). Bisphosphonate-related osteonecrosis of the jaw: Awareness and level of knowledge of Lebanese physicians. Support. Care Cancer..

[28] De Lima P.B., Brasil V.L., de Castro J.F., de Moraes Ramos-Perez F.M., Alves F.A., dos AnjosPontual M.L., da Cruz Perez D.E. (2015). Knowledge and attitudes of Brazilian dental students and dentists regarding bisphosphonate-related osteonecrosis of the jaw. Support. Care Cancer..

[29] Alhussain A., Peel S., Dempster L., Clokie C., Azarpazhooh A. (2015). Knowledge, practices, and opinions of Ontario dentists when treating patients receiving bisphosphonates. J. Oral Maxillofac. Surg..

[30] Bedogni A., Fedele S., Bedogni G., Scoletta M., Favia G., Colella G., Agrillo A., Bettini G., Di Fede O., Oteri G. (2014). Staging of osteonecrosis of the jaw requires computed tomography for accurate definition of the extent of bony disease. Br. J. Oral Maxillofac. Surg..

[31] Fedele S., Bedogni G., Scoletta M., Favia G., Colella G., Agrillo A., Bettini G., Di Fede O., Oteri G., Fusco V. (2015). Up to a quarter of patients with osteonecrosis of the jaw associated with antiresorptive agents remain undiagnosed. Br. J. Oral Maxillofac. Surg..

[32] Wilde F., Heufelder M., Lorenz K., Liese S., Liese J., Helmrich J., Schramm A., Hemprich A., Hirsch E., Winter K. (2012). Prevalence of cone beam computed tomography imaging findings according to the clinical stage of bisphosphonate-related osteonecrosis of the jaw. Oral Surg. Oral Med. Oral Pathol. Oral Radiol..

[33] Setabutr D., Hales N.W., Krempl G.A. (2010). Necrotizing fasciitis secondary to bisphosphonate-induced osteonecrosis of the jaw. Am. J. Otolaryngol..

[34] Randi L., De Martino I., Fasciolo A., Gambino A., Fusco V. (2014). Osteonecrosis of jaw (ONJ): Sometimes a life-threatening event. Literature review and two cases. Ann. Stomatol. (Roma).

[35] Mondello P., Pitini V., Arrigo C., Mondello S., Mian M., Altavilla G. (2014). Necrotizing fasciitis as a rare complication of osteonecrosis of the jaw in a patient with multiple myeloma treated with lenalidomide: Case report and review of the literature. SpringerPlus.

[36] Yang R.H., Shen S.H., Li W.Y., Chu Y.K. (2015). Bisphosphonate-related osteonecrosis of the jaw complicated by Ludwig’s angina. J. Chin. Med. Assoc..

[37] Mehanna P., Goddard R. (2010). Bisphosphonate associated osteonecrosis: An unusual case. Aust. Dent. J..

[38] Ebker T., Rech J., von Wilmowsky C., Neukam F.W., Stockmann P. (2013). Fulminant course of osteonecrosis of the jaw in a rheumatoid arthritis patient following oral bisphosphonate intake and biologic therapy. Rheumatology (Oxf.).

[39] Kaehling C.H., Streckbein P., Schmermund D., Henrich M., Burchert D., Gattenloehner S., Howaldt H.P., Wilbrand J.F. (2014). Lethal cervical abscess following bisphosphonate related osteonecrosis of the jaw. J. Craniomaxillofac. Surg..

[40] Qaisi M., Hargett J., Loeb M., Brown J., Caloss R. (2016). Denosumab Related Osteonecrosis of the Jaw with Spontaneous Necrosis of the Soft Palate: Report of a Life Threatening Case. Case Rep. Dent..

[41] Otto S., Schreyer C., Hafner S., Mast G., Ehrenfeld M., Stürzenbaum S., Pautke C. (2012). Bisphosphonate-related osteonecrosis of the jaws—Characteristics, risk factors, clinical features, localization and impact on oncological treatment. J. Craniomaxillofac. Surg..

[42] Di Fede O., Bedogni A., Giancola F., Saia G., Bettini G., Toia F., D’Alessandro N., Firenze A., Matranga D., Fedele S. (2016). BRONJ in patients with rheumatoid arthritis: A multicenter case series. Oral Dis..

[43] Vescovi P. (2012). Bisphosphonates and osteonecrosis: An open matter. Clin. Cases Miner. Bone Metab..

[44] Walter C., Sagheb K., Bitzer J., Rahimi-Nedjat R., Taylor K.J. (2014). Analysis of reasons for osteonecrosis of the jaws. Clin. Oral Investig..

[45] Papapoulos S., Lippuner K., Roux C., Lin C.J., Kendler D.L., Lewiecki E.M., Brandi M.L., Czerwiński E., Franek E., Lakatos P. (2015). The effect of 8 or 5 years of denosumab treatment in postmenopausal women with osteoporosis: Results from the FREEDOM Extension study. Osteoporos. Int..

[46] Sánchez A., Blanco R. (2017). Osteonecrosis of the jaw (ONJ) and atypical femoral fracture (AFF) in an osteoporotic patient chronically treated with bisphosphonates. Osteoporos. Int..

[47] Bagan J., Peydró A., Calvo J., Leopoldo M., Jiménez Y., Bagan L. (2016). Medication-related osteonecrosis of the jaw associated with bisphosphonates and denosumab in osteoporosis. Oral Dis..

[48] European Medicines Agency (EMA) (2015). PSUR/PSUSA Submission and Expected PRAC Outcome Dates for Bisphosphonates and Denosumab. http://www.ema.europa.eu/docs/en_GB/document_library/Other/2015/03/WC500184239.pdf.

